# Maternal Depression Affects Infants’ Lexical Processing Abilities in the Second Year of Life

**DOI:** 10.3390/brainsci10120977

**Published:** 2020-12-12

**Authors:** Ruth Brookman, Marina Kalashnikova, Janet Conti, Nan Xu Rattanasone, Kerry-Ann Grant, Katherine Demuth, Denis Burnham

**Affiliations:** 1 The MARCS Institute for Brain, Behaviour and Development, Western Sydney University, Locked Bag 1957, Penrith, NSW 2750, Australia; m.kalashnikova@bcbl.eu (M.K.); denis.burnham@westernsydney.edu.au (D.B.); 2 School of Psychology, Western Sydney University, Locked Bag 1957, Penrith, NSW 2750, Australia; j.conti@westernsydney.edu.au; 3 Basque Centre for Cognition, Brain and Language, Paseo Mikeletegi 69, 2º, 20009 Donostia-San Sebastián, Spain; 4 IKERBASQUE, Basque Foundation for Science, Plaza Euskadi 5, 48009 Bilbao, Spain; 5 Department of Linguistics, Macquarie University, Balaclava Road, North Ryde, NSW 2109, Australia; nan.xu@mq.edu.au (N.X.R.); Katherine.demuth@mq.edu.au (K.D.); 6 Health Education and Training Institute, Locked Bag 7118, Parramatta Bc, NSW 2124, Australia; Kerry.ann.grant@gmail.com

**Keywords:** language development, lexical processing, vocabulary, postnatal, maternal depression, maternal anxiety, infancy

## Abstract

Maternal depression and anxiety have been proposed to increase the risk of adverse outcomes of language development in the early years of life. This study investigated the effects of maternal depression and anxiety on language development using two approaches: (i) a categorical approach that compared lexical abilities in two groups of children, a risk group (mothers with clinical-level symptomatology) and a control non-risk group, and (ii) a continuous approach that assessed the relation between individual mothers’ clinical and subclinical symptomatology and their infants’ lexical abilities. Infants’ lexical abilities were assessed at 18 months of age using an objective lexical processing measure and a parental report of expressive vocabulary. Infants in the risk group exhibited lower lexical processing abilities compared to controls, and maternal depression scores were negatively correlated to infants’ lexical processing and vocabulary measures. Furthermore, maternal depression (not anxiety) explained the variance in infants’ individual lexical processing performance above the variance explained by their individual expressive vocabulary size. These results suggest that significant differences are emerging in 18-month-old infants’ lexical processing abilities, and this appears to be related, in part, to their mothers’ depression and anxiety symptomatology during the postnatal period.

## 1. Introduction

An infant’s developmental trajectory is shaped by both environmental and genetic factors [1]. Communication with a primary caregiver, usually the mother, is an infant’s first social and linguistic experience and exerts a strong influence on the infant’s language development [2,3]. However, infancy is also associated with an increased risk of maternal depression and anxiety, the two most common emotional health conditions experienced by mothers after giving birth, e.g., [4,5]. Postnatal depression and anxiety can have long-lasting effects on children’s socio-emotional development, e.g., [6,7]. Recent research suggests that children’s cognitive development can be impacted in the first years of life. Specifically, in the domain of language acquisition, recent studies have shown negative effects of maternal depression and anxiety on infants’ expressive vocabulary skills at 12 and 18 months of age [8,9]. Importantly, findings suggest that effects may be dependent on the severity of maternal depression and anxiety symptoms and are not restricted to infants of mothers with clinical levels of these disorders [10]. This study investigates the relation between maternal depression and anxiety at both clinical and subclinical levels and infants’ lexical development by including an assessment of infants’ expressive vocabulary size as well as an objective measure of receptive vocabulary and efficiency of lexical processing, which have not been investigated in this population.

### 1.1. Maternal Depression and Anxiety and Links with Language Development

One-quarter to one-third of women in Western societies experience one major depressive episode in their lifetime [11]. In clinical settings, health professionals frequently use the term postnatal depression (PND) to refer to depression symptoms experienced in the postnatal period that are similar to those of major depressive disorder (MDD). According to the Diagnostic and Statistical Manual of Mental Disorders, Fifth edition (DSM-5: [12]), PND is referred to as a specifier of MDD, with symptoms including a loss of appetite, sleep disturbance, weight changes, loss of interest in pleasurable activities, agitation, concentration difficulties, feelings of guilt and worthlessness, and suicidal thoughts. In addition to depression, there is a high incidence of anxiety disorders in this population, with prevalence estimates ranging from 3% to 43% in Western societies [13]. The DSM-5 notes that anxiety disorders can include, for example, generalized anxiety, specific phobias, and panic disorders [12]. In a similar manner to postnatal depression, postnatal anxiety symptoms can lead to a significant impairment in daily functioning [14]. Importantly, these two conditions often co-occur in the postnatal period [15], and it is not always possible to isolate their independent effects on maternal mental health and possible subsequent effects on infants’ cognitive development. For this reason, this study considers the two conditions together.

There is growing evidence that maternal depression can yield early and long-lasting effects on children’s linguistic development. The National Institute of Child Health and Human Development (NICHD) Early Child Care Research Network conducted a longitudinal study that followed mother–infant dyads from six to 15, 24, and 36 months, and assessed children’s language and general cognitive abilities at 36 months [16]. After controlling for demographic risk factors, children of mothers with chronically or occasionally elevated depression scores performed less optimally compared to children of non-depressed mothers on measures of verbal comprehension and expressive language in addition to an assessment of school readiness. More recently, Kaplan et al. [10] found a significant negative correlation between self-reported maternal depression scores and 12-month-old infants’ percentile scores on the Bayley Expressive Communication subscale but not the general cognitive and receptive communication subscales of the Bayley-III [17]. A similar study focusing on maternal anxiety examined the emerging language abilities of 12-month-old infants with mothers who met the criteria for an anxiety disorder in the postnatal period [9]. This study showed that infants scored significantly lower than controls in the language subtests of the Bayley-III [17]. Taken together, the above studies suggest that the onset of language delay in the children of mothers affected by depression or anxiety may commence quite early during infancy and be reflected in infants’ early pre-lexical and lexical skills.

Overall, compared to maternal depression, there is a scarcity of research examining the link between maternal anxiety and adverse developmental findings [13]. Inconsistencies in findings from previous studies examining maternal anxiety have been attributed to the lack of homogeneity in defining and measuring anxiety and its high co-morbidity with depression [13]. Furthermore, postnatal depression symptoms may mask anxiety symptoms, leading to a risk of postnatal anxiety disorders remaining undetected and untreated [18,19]. In this study, we assess both maternal depression and anxiety. Our analyses consider these conditions together for the purpose of classifying mothers into groups of those who are and are not affected by a depression and/or anxiety diagnosis, and also separately, to measure the relation of each condition to infants’ early language development.

### 1.2. Language Learning and Social–Environmental Influences

The observed delays in language outcomes have been attributed to the effects of maternal mental health concerns on the quality of infants’ early linguistic environment [8]. This view emphasizes the important role that infants’ early environments and socio-communicative interactions have in supporting their language development. It also posits that language learning is socially mediated by caregiver interactions with their children [20]. Accordingly, it is said that mothers support their infants’ communication development through the quality and quantity of their speech input [21], both of which can contribute positively to a rich and stimulating home language environment [22]. This supporting role, however, may be challenged by the presence of maternal mental health concerns affecting the quality and quantity of day-to-day interactions with the infant and leading to long-lasting effects in the infant’s development.

A recent study by Brookman et al [8] provides direct evidence for the proposal that adverse outcomes in infants’ language development are due to the disruptions to the quantity of the mother–infant interaction related to maternal depression [23]. This longitudinal study examined the home language environments of a group of infants at risk for language delays by virtue of having mothers affected by emotional health concerns, compared with a group of not-at-risk controls. Analyses of day-long audio recordings conducted when the infants were six and 12 months old revealed that at-risk infants were exposed to significantly fewer conversational turns and vocalized significantly less than controls. Importantly, over and above maternal depression and anxiety measures, individual variations in infants’ early home language environment (conversational turns and vocalization counts) predicted infants’ expressive vocabulary size at 18 months.

From these studies, it can be concluded that mothers who speak more to their infants tend to have children who develop more advanced language abilities, e.g., [24,25], and that this is the case for mothers who do and do not exhibit clinical levels of mental health concerns. Moreover, research suggests that the effects of frequency of mother–infant interactions can be observed not only on children’s developing vocabulary size but also on lexical processing efficiency—infants’ ability to access the meaning of familiar words stored in the lexicon. Weisleder and Fernald [26] assessed the quantity of early language input and lexical processing efficiency in children from low and high socio-economic status (SES) backgrounds. Differences emerged at 18 months between SES groups in both vocabulary development and language processing efficiency. In addition, the effect of the amount of speech input on expressive vocabulary was mediated by language processing efficiency, suggesting that exposure to richer or higher-quality speech input supports infants’ processing abilities and, therefore, their vocabulary growth.

Infants’ lexical processing efficiency abilities undergo a significant change in their second year of life, whereby infants become increasingly fast, more accurate, and more efficient at recognizing familiar words in continuous speech and in different contexts [27,28,29]. Individual differences observed in this process are developmentally meaningful as they predict individual vocabulary growth and the development of more advanced linguistic abilities in early childhood and even school age [29]. Word recognition skills demonstrated by toddlers at 25 months of age have been found to predict later language skills at eight years of age [30]. In addition, individual differences in processing speed in pre-term infants tested at 18 months of age were shown to predict receptive vocabulary at 3 years [31] and global language and cognitive abilities at 4.5 years [32]. Infants’ lexical processing abilities have been found to have high predictive validity for later literacy skills, school readiness, and academic performance in primary school, e.g., [33]

Lexical processing efficiency is typically assessed using Looking-While-Listening (LWL) paradigms and is an important research concept when exploring individual differences in infants’ language development [34]. Infants’ gaze patterns are recorded while infants hear familiar words and look at referents on a screen that do or do not correspond to these words (e.g., hearing the phrase “look at the apple” while seeing a picture of an apple and a shoe on a screen). This procedure uses real-time eye-tracking measures of children’s gaze patterns in response to speech and imposes low cognitive demands, which makes it well suited for infants in their second year of life, in particular here, for objectively measuring young infants’ lexical competence. Previous research involving infants of mothers affected by depression has primarily included language measures based on standardized IQ batteries and parental reports [8,10], both of which can be problematic when used with populations at risk for developmental language delays. For example, while parental reports are a well-established method for obtaining valuable information about infants’ early language development [35], they can be vulnerable to parents’ under- or over-estimations of their child’s abilities [36]. The LWL procedure provides a valuable objective assessment and, thus, complements parental reports of vocabulary size [26]. The LWL procedure can provide a more detailed picture of young infants’ ability to comprehend or find meaning in spoken language (see [37]).

### 1.3. The Present Study

This study investigated the speech processing efficiency and vocabulary size of infants whose mothers have depression and anxiety symptoms. The study included a subset of mother–infant dyads from the longitudinal cohort from Brookman et al [8]. Infants’ vocabulary size and lexical processing abilities were assessed when they were 18 months old, a time that is associated with the “vocabulary spurt”, e.g., [24]. Infants’ vocabulary size was assessed using a parental report and lexical processing was assessed using the LWL procedure.

Maternal depression and anxiety were measured at multiple time points in the postnatal period. This enabled the employment of two complementary analytical approaches. First, we employed a categorical approach by allocating mother–infant dyads to a risk or no-risk control group. The aim of this was to assess the differences in early language abilities (lexical processing and vocabulary size) between infants whose mothers were affected by clinical levels of symptomatology of either depression, anxiety, or both versus a no-risk control group. Mothers in the risk group had a current diagnosis of depression and anxiety and/or elevated symptoms (reaching a clinical threshold) reported in the postnatal period. Second, we employed a continuous approach, in which the aim was to assess the relationship between individual differences in the severity of maternal depression and anxiety symptoms on the one hand and infants’ language outcomes on the other. In this case, data from the entire sample were collapsed, placing mothers on a continuum of depression and anxiety measures and assessing the predictive validity of levels of emotional health concerns on infants’ language abilities.

Two predictions were tested. First, it was predicted that the categorical approach comparisons would reveal deficits in lexical processing abilities and lower expressive vocabulary sizes in infants in the risk group compared with infants in the control group, e.g., [9]. Second, it was predicted that the correlational and regression analyses of the continuous approach would demonstrate that infants’ speed and accuracy of lexical processing and their expressive vocabulary size would be correlated with maternal depression and anxiety scores. That is, the effects of maternal depression and anxiety symptoms were predicted to be incremental with subclinical expression and not only present in families where the mother displays clinical levels of depression or anxiety symptomatology, e.g., [8,10].

## 2. Materials and Methods

### 2.1. Participants

Forty-six mother–infant dyads (24 female and 22 male infants) participated. Lexical processing data for 10 infants were excluded from the final analysis due to equipment failure (*n* = 2), an acquired hearing loss (*n* = 1), failure to contribute sufficient gaze data for analyses (see Data processing in Section 2.3.1 for details, *n* = 2), and failure to complete the task (*n* = 5). The remaining 36 infants (17 female and 19 male) were in the risk group (*n* = 17) or the control group (*n* = 19) based on the mothers’ current diagnosis or elevated symptoms of depression and anxiety (see Section 2.2 Maternal Measures below). All infants were born into households with two heterosexual parents, full term (37–42 weeks), with normal birth weight, and no history of birth or postnatal complications. The infants were acquiring English in a monolingual context and were not exposed to a second language, and they had no reported hearing difficulties, neurological conditions, or health problems.

At the time of recruitment, mothers’ ages ranged from 26 to 41 years (Mean [*M*] = 33, Standard Deviation [*SD*] = 4.05). Maternal education levels for all mothers ranged from high school to postgraduate degree. A comparison between groups indicated that mothers from the risk group had significantly higher education levels than controls; Mann–Whitney *U* = 103, *p* = 0.040. This difference was due to more at-risk mothers having postgraduate degrees (Risk = 7 > Control = 5) while more control mothers had undergraduate degrees (Control = 14 > Risk = 12). Nevertheless, the median education level of both groups was an undergraduate university degree. Household income was also assessed by using the estimated average weekly household income levels based on participating families’ place of residence (Australian Bureau of Statistics). There was no significant group difference in estimated household income levels; Mann–Whitney *U* = 106, *p* = 0.051. See Table 1 for descriptive and inferential statistics of participants’ demographic information.

Initial recruitment for this study was conducted by contacting a community sample of mothers who were previously enrolled in a large-scale longitudinal project examining the effects of prenatal anxiety on infants’ development and cortisol levels at 12 weeks postpartum, and whose infants were within the correct age range for this study. This sample included mothers who did and did not have elevated depression and anxiety scores. Fourteen mother–infant dyads were recruited using this method. The remaining mother–infant dyads were recruited from an infant laboratory database and via the distribution of study flyers through community notice boards, libraries, playgroups, community agencies, and social media. This study was approved by the Western Sydney University Human Research Ethics Committee (approval number: H11703). All mothers provided written informed consent prior to participating in the study.

### 2.2. Maternal Measures

Given the high comorbidity of depression and anxiety, all mothers were administered measures of both conditions. The Center for Epidemiologic Studies Depression Scale—Revised (CESD-R) [38] was used to measure depression symptoms. The CESD-R is a 20-item self-reported scale of depressive symptoms and has been used widely with maternal populations in the perinatal period [39]. It has excellent psychometric properties, including strong factor loadings, high internal consistency, and theoretically consistent convergent and divergent validity [40]. The State Scale of the State-Trait Anxiety Inventory (STAI) [41] is a reliable and valid measure of current self-reported anxiety that is one of the most commonly used measures to capture clinical and subclinical anxiety levels [41,42]. The STAI has demonstrated good internal consistency in prior Australian studies with child-bearing women, e.g., [18].

Mothers commenced the study when their infants were six months old. To account for the potential variation in the severity and persistence of symptoms of maternal emotional health concerns during the postnatal period, which is known to influence child development outcomes, mean postnatal depression scores and mean postnatal anxiety scores (six, nine, 12, and 18 months) were calculated for each participant by averaging scores obtained at each data collection point (see Supplementary Materials Table S1, for details). Mothers were allocated to the risk group if they had (i) a self-reported current diagnosis of depression or anxiety; or (ii) depression and/or anxiety scores of which either or both exceeded the clinical threshold (i.e., CESD-R ≥ 16; STAI ≥ 40) at any of the four testing time points. The remaining mothers were allocated to the control group. Mother–infant dyads remained in the risk group for the duration of the study despite potential changes to their emotional health scores, due to evidence that impaired mother–infant interactions styles can persist beyond the remission of depression with ongoing risks to infant developmental outcomes, e.g., [43].

### 2.3. Infant Measures

#### 2.3.1. Lexical Processing

Stimuli. Six target words (ball, book, car, cup, hat, and shoe) were selected on the basis of previous research and familiarity to 18-month-old infants [29,44]. An Australian-English-speaking female was recorded producing the target words embedded in the carrier phrases “Where is the [target]?” and “Look at the [target]” in a lively, child-directed manner. In order to verify that the stimuli were comparable to other studies, an acoustic analysis of the phrases was conducted using Praat, version 6.0.40 [45] (see Supplementary Materials Table S2, for details). Results indicated that pitch and word duration were in the usual range for infant-directed speech (IDS) and consistent with stimuli used in previous research [46,47,48].

**Figure 1:**
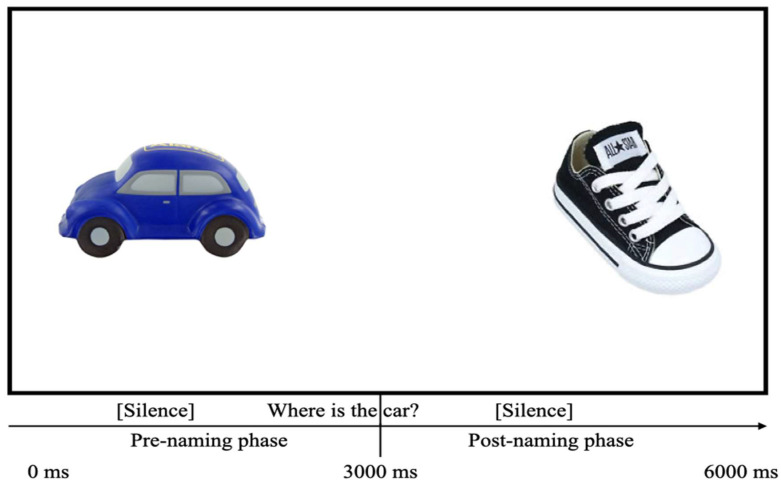
Structure of a sample trial from the lexical processing looking-while-listening (LWL) paradigm.

Visual stimuli for test trials consisted of 6 colorful pictures of familiar target objects that were presented in pairs placed side-by-side (target and distracter). The pictures were presented on a white background and selected to be comparable in size and visual salience. The images appeared in blocked pairs: ball–cup, hat–book, and shoe–car. The audio and visual stimuli were combined into video clips containing 24 test trials using iMovie software version 10.0.9 (Apple Inc.). Each clip’s duration was 6 s, and it was sub-divided into two 3-s phases: pre-naming and post-naming (Figure 1).

Procedure. Infants’ lexical processing skills were assessed using the LWL procedure (see [37]). Infants sat on their mother’s lap approximately 60 cm away from a 19-inch square LG monitor and listened to auditory stimuli presented through an Edirol MA-15D speaker (Roland Corporation) placed directly below the monitor. Mothers listened to mixed auditory sounds via headphones to prevent them from interfering with the infants’ performance. In addition, mothers were instructed to divert their eyes away from the computer monitor to prevent their own gaze from interfering with the eye-tracker’s recording, and they were asked to avoid pointing to the screen or talking to their infant during the task. Infants’ gaze duration and direction were recorded using a Tobii X120 eye tracker(Tobii Technology AB). A researcher observed the infant from an adjoining control room via a live feed using a webcam located on the top of the computer monitor and directed towards the infant’s face.

Prior to beginning the task, infants completed a five-point infant calibration routine. Next, infants proceeded to the test, which consisted of 24 test trials and 4 filler trials [49]. The 24 test trials were divided into 4 blocks. Each block consisted of six trials, one for each target word. Infants were, therefore, exposed to each target word 4 times. The order of trials within each block was randomized. In two blocks, the target words were presented using the carrier phrase “Where is the (target)?”, and in two blocks, they were presented using the phrase “Look at the (target)!”. In each block, the target picture was presented an equal number of times on the left and right side. Before the commencement of each trial, infants were presented with an attention-getter stimulus. The presentation of each trial was controlled by the researcher upon the infant fixating their gaze on the attention-getter stimulus. Between each block, the infants were presented with a filler trial aimed to maintain their attention to the screen, which displayed the images of four familiar images (that were not part of the test trials) accompanied by the exclamations ‘wow’ and ‘look’ recorded by the same speaker. The total duration of the task was approximately five minutes.

Data processing. Two rectangular areas of interest (AoIs) encompassing each object were defined for each test trial. The AoIs were located on the right and left sides of the screen encompassing the visual referent. The size and position of the AoIs were identical across all trials.

Raw gaze data were extracted from Tobii Studio software and were processed using Eye Tracking R in R [50]. First, segments with data loss were identified, and trials with less than 25% detected gaze were excluded from all subsequent analyses. On average, infants contributed trials with 72% of detected gaze per trial, and this did not differ between the risk and the control groups, *t*(29) = −0.321, *p* = 0.750. Next, two response windows were defined: the pre-naming window and the post-naming window. The pre-naming window was from 0 to 3300 ms which included looks to the target and the distracter prior to the presentation of the target label. The post-naming window is the critical response window for analysis of the accuracy and latency of infants’ looking behaviors. This window was from 3300 to 4800 ms, which included looks to the target and the distracter in response to the target label. A 1500-ms response window was used for analyses as looking behaviors later in the trial are unlikely to represent responses to the target label [29]. Proportion of time looking (PTL) to the target out of the total looking time to the target (T) and the distracter (D) was calculated [PTL = T/(T + D)] for each time window and was used as the dependent variable in all analyses.

Finally, trials were categorized based on whether the infant had fixated the target object (target-initial trials) or distracter object (distracter-initial trials) at the onset of the target word [37]. This enabled an onset contingent analysis to assess the latency (response time) required to switch gaze from distracter to target object after hearing the target name.

#### 2.3.2. Expressive Vocabulary Checklist

At 18 months of age of the children, their mothers completed the Australian English (OZI) [51] adaptation of the MacArthur–Bates Communicative Inventory [52]. Mothers were asked to identify words on the checklist that their child could produce. OZI data were unavailable for two infants due to the mother’s failure to complete the OZI.

## 3. Results

First, our categorical approach employed independent *t*-tests to compare groups on accuracy and latency scores to test our first prediction that infants’ lexical processing abilities and expressive vocabulary sizes would be lower in the risk group compared with the control group. Further, one-sample *t*-tests were conducted to compare group accuracy scores against chance levels. Second, our continuous approach employed a correlational analysis to examine the extent to which mean depression and anxiety scores were correlated with infant accuracy and latency scores. Next, hierarchical regression analyses were used to test our second prediction that infants’ speed and accuracy of lexical processing, and their expressive vocabulary size, would be negatively correlated with maternal depression and anxiety scores.

### 3.1. Lexical Processing

**Figure 2:**
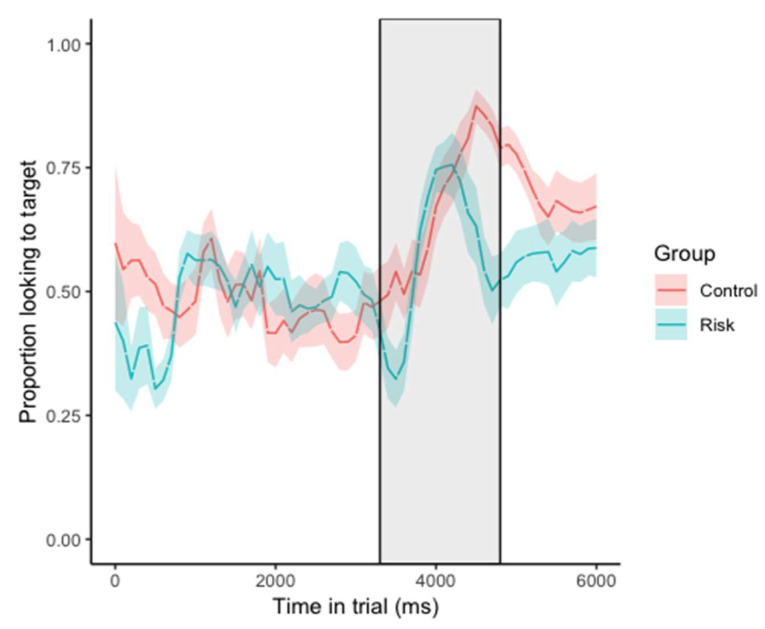
Time course plot of the proportion of looking time to the target across lexical processing trials (samples taken every 100 ms; shading around the line represents the 95% confidence intervals). Shaded rectangle area represents the post-naming analysis window.

The time course of looking behaviors by the control and risk infants during the experimental trials is presented in Figure 2. First, infants’ PTL to the target during the pre-naming window was compared between the control and risk groups to ensure that infants in the two groups did not differ in their baseline gaze patterns to the two objects before the onset of the target label. An independent samples *t*-test showed no significant group differences; *t*(30) = −0.306, *p* = 0.762, *d* = −0.112. Furthermore, one-sample *t*-tests confirmed that infants in the control, *t*(17) = −0.872, *p* = 0.395, *d* = −0.209, and risk groups, *t*(13) = −0.272, *p* = 0.790, *d* = −0.074, did not look at the targets significantly above chance levels (chance = 0.5) prior to hearing its label. These analyses demonstrate that infants in the two groups did not exhibit any visual preferences to the referents used in the task that may have impacted their post-naming looking behaviors.

Next, post-naming performance was compared between the two groups on measures of accuracy and latency upon hearing the target label.

#### 3.1.1. Accuracy

**Figure 3:**
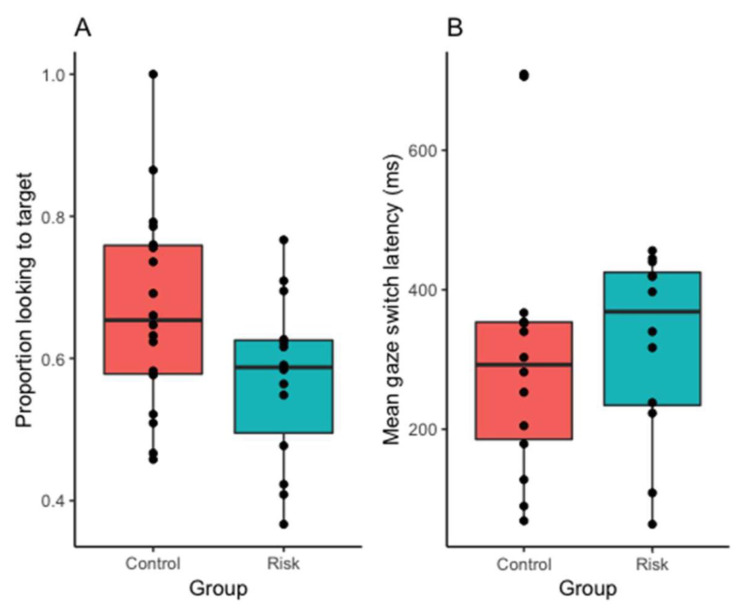
Mean accuracy (**A**) and latency (**B**) of infants’ lexical processing performance (black dots represent individual scores and the internal line represents the median, and the hinges extend to the first and third quartiles).

The term accuracy refers to infants’ proportions of correct fixation (i.e., the proportion of looking to the target versus distracter following hearing the target label). An independent samples *t*-test showed that infants in the control group were significantly more likely to fixate the target than infants in the risk group after hearing its label; *t*(30) = 2.085, *p* = 0.046, *d* = 0.761. One-sample *t*-tests were conducted to determine whether infants were fixating the target above chance levels (0.5) (a Bonferroni correction was used to adjust the *p*-value to 0.025 to account for multiple comparisons). Infants in the control group fixated the target significantly above chance levels, *t* (17) = 5.082, *p* < 0.001, *d* = 1.181, but this was not the case for the risk group, *t*(13) = 2.265, *p* = 0.041, *d* = 0.602 (Figure 3).

#### 3.1.2. Latency

The term latency refers to the measure of the speed with which infants switched their gaze from the distracter to target after hearing the target label. In order to access infants’ lexical processing efficiency, an onset contingent gaze analysis was conducted. Only trials in which the infant was fixating the distracter at the start of the post-naming phase were included in this analysis in order to assess the latency of gaze switches from the distracter to the target in response to the target label. Twenty-two infants did not contribute data to these analyses, and data for four infants were excluded as their latency values were identifies as outliers (greater than 3 SD away from the mean). Raw latencies were converted from msec to z-scores for these analyses. An independent samples *t*-test showed that infants in the risk group were marginally slower in directing their gaze to the target compared to the control infants, but this did not reach statistical significance; *t* (20) = −1.927, *p* = 0.068. *d* = −0.862.

### 3.2. Expressive Vocabulary Size

No statistically significant differences were observed in the expressive vocabulary scores for the risk and control groups, *t*(29) = 0.806, *p* = 0.427, *d* = 0.216, even though control infants’ scores (*M* = 83.72, *SD* = 48.16) were numerically higher compared to the risk infants (*M* = 65; *SD* = 80.91).

### 3.3. Links between Infants’ Lexical Abilities and Maternal Depression and Anxiety Measures

The next set of analyses from our continuous approach includes data collapsed from the entire sample with depression and anxiety measures used as continuous variables. An inspection of the data revealed that maternal depression and anxiety scores and infants’ vocabulary size were not normally distributed, so Spearman correlation analyses were employed. As can be seen in the results reported in Table 2, correlation analyses were conducted between the infant measures of lexical abilities and mean maternal depression and anxiety measures. As hypothesized, infants’ expressive vocabulary size at 18 months was significantly correlated to the proportion of looking time to target post-naming, ρ(31) = 0.594, *p* = 0.000, and latency of gaze switch to target, ρ(21) = −0.609, *p* = 0.003. This confirms that our experimental task was capturing individual lexical abilities. Importantly, infants’ lexical processing accuracy was also significantly correlated to mean maternal depression, ρ(32) = −0.538, *p* < 0.001, and mean maternal anxiety, ρ(32) = −0.434, *p* = 0.013. Infants of mothers with higher mean depression and anxiety scores in the postnatal period were less likely to fixate the target object after hearing its label in this task. Latency was not significantly correlated with mean depression and anxiety scores.

**Figure 4:**
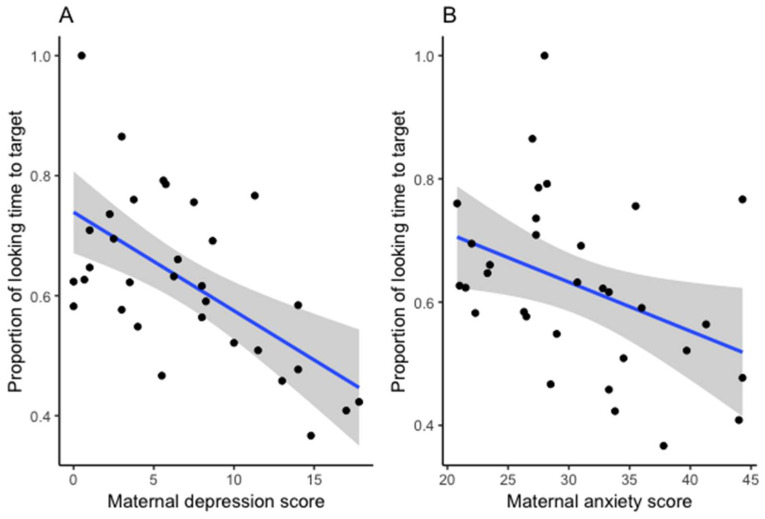
Infants’ lexical processing accuracy scores in relation to maternal depression (**A**) and anxiety (**B**) (the lines illustrate the linear model results and shaded areas represent the standard error of the mean).

Next, two hierarchical linear regression models were constructed to assess whether maternal depression and anxiety explained variance in infants’ individual lexical processing performances above the variance explained by their individual expressive vocabulary size. The first model included proportion of looking time to target post-naming as the dependent variable, and the second model included latency of gaze switch to target as the dependent variable. Both models removed variance of the expressive vocabulary scores in the first block and included mean depression and anxiety scores as the predictor variables in the second block. Both models were significant in accounting for 34.6% and 20.7% of variance, respectively. However, maternal depression was the only significant predictor of infants’ proportion of looking time to target in the lexical processing task in Model 1 but not in Model 2 (see Table 3). As illustrated in Figure 4, after removing the variance explained by vocabulary size, maternal depression remained a significant predictor of lexical processing performance, but this was not the case for maternal anxiety.

## 4. Discussion

This is the first study to investigate the lexical processing abilities and expressive vocabulary size in infants whose mothers have elevated depression and anxiety symptoms. Consistent with our predictions, the categorical approach revealed that infants in the risk group demonstrated deficits in their lexical processing abilities. Further, our continuous approach revealed that infants’ lexical processing abilities are correlated with their mothers’ depression scores. Contrary to our predictions, at-risk infants’ expressive vocabulary sizes did not differ from controls, but a negative correlation was found between infants’ expressive vocabulary size and maternal depression scores. Finally, maternal depression (not anxiety) explained variance in infants’ individual lexical processing performance above the variance explained by their individual expressive vocabulary sizes.

First, this study aimed to examine the effects of maternal depression and anxiety on infants’ performance on a lexical processing task. One of the most important findings of this study is the observation that mothers’ depression and anxiety symptoms have significant effects on their 18-month-olds’ lexical abilities. That is, infants in the risk group did not identify the target above chance levels. It is noted that the risk group did perform above chance when the strict Bonferroni correction was not applied, but importantly, their performance was significantly different to the performance of infants in the control group. Therefore, infants in the risk group demonstrated significantly poorer ability to identify the referents of spoken words compared to same-age controls.

While this is the first study to examine the effect of maternal depression on lexical processing abilities, our findings are consistent with the previously reported links between maternal depression and infants’ lexical competence, e.g., [8,10]. Our results extend the previous literature by showing that deficits in lexical abilities, specifically lexical processing efficiency, are present early in infancy. Furthermore, maternal depression in the postnatal period may impact not only infant vocabulary size but also how infants access information within their vocabularies. Most importantly, this finding demonstrates that deficits identified in later language skills are not only associated with vocabulary size but with foundational processes related to language acquisition, and that these associations can be detected in children’s second year of life.

The second aim of this study consisted in examining the effect of maternal depression and anxiety on infants’ lexical processing skills and expressive vocabulary size at the full sample level. It was predicted that infants’ accuracy and speed of lexical processing would be correlated with maternal depression and anxiety scores. This was the case for accuracy and depression and anxiety scores as discussed above, but latency was not correlated with either of the emotional health variables. The response time required for the risk-group infants to switch from distracter to target was only marginally slower than that of infants in the control group. It is likely that the absence of a group difference here for latency is due to the smaller sample size (*n* = 22) caused by the exclusion of infants who did not contribute any distracter-initial trials. However, the absence of a link between latency and vocabulary size has been observed in another lexical processing study, which yielded a relation between accuracy and vocabulary scores, but not for latency and the vocabulary scores of 18-month-old infants [53]. A positive correlation between latency and vocabulary size was evident, however, when the same infants were tested again at 24 months. Therefore, additional research with latency data from a larger sample (e.g., by using adaptations of the LWL to maximize the number of distracter-initial trials [54]) and from different age groups is required before reliable conclusions about the effects of maternal emotional health concerns on infants’ lexical processing efficiency can be reached.

Consistent with our prediction, depression scores were negatively correlated with vocabulary size. This finding is consistent with several outcome studies that have associated maternal depression with delays in 12-month-old children’s language outcomes [10]. Furthermore, Milgrom et al. [55] assessed 42-month-old infants with mothers who had been admitted as inpatients for major depression during the perinatal period. They found that these infants had significantly lower language and cognitive scores on the Early Screening Profile compared to controls. In addition, they had lower scores on the full scale (but not verbal IQ) of the Wechsler Preschool and Primary School Scale of Intelligence—Revised (WPPSI-R). Interestingly, these outcomes were mediated by observer-based assessments of maternal responsiveness to their infants (the ability to respond promptly, contingently, and appropriately to infants’ communication cues) when they were at six months of age.

The absence of a relationship with maternal anxiety scores and vocabulary in the present study is not consistent with recent findings that 12-month-old infants with mothers with an anxiety disorder score significantly lower than controls on standardized measures of their language abilities [9]. Furthermore, after removing the variance explained by vocabulary size, our findings specified maternal depression (and not anxiety) as a significant predictor of lexical processing performance. This finding is consistent with empirical evidence that identifies maternal depression to be a consistent predictor of adverse developmental outcomes and maternal anxiety to be a less consistent predictor [13]. In our study, the risk group was defined by measures of depression and anxiety taken together in order to reflect the high degree of comorbidity between the two conditions. However, future research with a larger sample size could consider direct comparisons between groups of mothers who have depression with those who have anxiety, as well as mothers who have co-morbid symptoms of both, as there is some evidence that comorbidity can be a marker of severity [56]. While our sample size was sufficient to reveal significant effects and was suitable for the statistical analyses reported here, a larger sample size would increase options for controlling for co-morbidity.

Our finding of an association between maternal emotional health symptoms in the postnatal period and lexical processing abilities in the second year of life of children is congruent with social constructionist views of language acquisition, which emphasize the importance of social interactions to support language learning [20]. There is evidence that depression can have a negative impact on these social interactions via both the quantity and quality of maternal speech to and interactions with infants. For example, the quantity of infant vocalizations and conversational turn counts can be adversely affected by maternal emotional health symptoms, which, in turn, predict infant vocabulary size at 18 months [8]. Our results extend this finding by revealing that infant lexical processing abilities are affected by the severity of maternal depression symptoms. It may be that a reduction in quantity of speech input in the risk group (vs. control group) contributed to the group difference in lexical processing abilities in the present study.

A reduction in the quality of speech input may provide a further explanation for the group differences in early lexical processing abilities observed here. As a special speech register, IDS is hypothesized to provide a rich environment for language learning for young infants [57]. It also has the potential to contribute to the pathway through which maternal depression influences child language outcomes [10], especially given that qualitative differences have been observed in the IDS produced by mothers with depression versus those without depression [58,59,60]. Research by Kaplan and colleagues [61] also links maternal depression to the quality of maternal IDS and infants’ performances in associative learning tasks. In a series of experiments using a conditioned-attention paradigm, IDS served as a priming stimulus that facilitated associative learning. In a later study, Kaplan et al. [62] found that the more monotonic IDS produced by mothers with depression was less effective in promoting basic learning by infants, such as when infants were required to learn associations between a face and a voice. Furthermore, older infants who had been exposed to this type of IDS for a longer period of time also demonstrated poorer learning in response to IDS produced by mothers without depression [63]. This suggests that infants have an experience-based change in their responsiveness to maternal IDS, which may alter their ability to learn from IDS cues from adults who are not impacted by depression. These known impacts of maternal depression on the language promoting benefits of IDS may have contributed to the group differences in lexical processing abilities observed here.

Our longitudinal design and the use of both categorical and continuous analytical approaches in this study enabled us to include mothers with subclinical symptoms of depression and anxiety in our analyses. This is important given the association between the severity of depression symptomatology (clinical and subclinical) and early language development [10]. Obtaining several measures allowed us to construct a profile of longer-term exposure to depression symptoms in the postnatal period as opposed to taking measures at single points in time. The chronicity of depression is known to influence expressive language scores, which are likely to be lower for children of chronically depressed mothers when compared to children of less depressed mothers [16]. Nevertheless, there are also several limitations of this study’s design that must be considered when interpreting our findings. First, our analyses are correlational, so it remains possible that alternative factors could account for the observed relations between maternal depression and anxiety and infants’ lexical processing abilities. In addition, this study included a largely homogeneous sample consisting of middle- or upper-middle-class families, where both parents were heterosexual and most mothers had higher education degrees. Therefore, it is critical for future research to assess these relations in more heterogenous samples to identify additional socio-economic factors that may influence maternal mental health and infants’ early cognitive development.

Despite the limitations outlined above, our findings point to several clinical implications for interventions to assist mother–infant dyads where infants may be at risk for adverse language outcomes. Empirical evidence indicates that anti-depressant medications and psychological interventions can assist in the reduction in maternal depression symptoms, e.g., [64,65]. These interventions, when given to mothers in isolation from infants, do not necessarily improve mother–infant interactions nor mitigate the risks to child development [66,67,68]. Psychoeducation and support, therefore, could be offered to families to assist in the facilitation of a rich home language learning environment for infants. In a parallel process to interventions with mothers experiencing depression, interventions with the mother–infant dyad could increase communication behaviors known to support language acquisition (see [69] for a review).

In summary, findings of this study highlight the negative effect of maternal depression and anxiety symptoms on infants’ early lexical processing abilities. Overall, these results suggest there are significant differences in 18-month-old infants’ lexical processing abilities related to their mothers’ depression symptomatology during the postnatal period. It is important for parents and health professionals working with infants to understand the real risk that maternal depression poses on children’s early language development. This knowledge would ensure that early intervention is delivered in a timely manner if such support is warranted.

## Figures and Tables

**Table 1:** Descriptive and inferential statistics for participants’ demographic information.

Mother and Infant Characteristics	Total Sample (*n* = 36)	Control Group (*n* = 19)	Risk Group (*n* = 17)
Infant Gender: *n* (%)			
Male Female	19 (53) 17 (47)	12 (63) 7 (37)	7 (41) 10 (59)
Birth weight (kg)			
Range *M* (*SD*)	2.38–4.89 3.49 (0.52)	2.38–4.74 3.38 (0.53)	3.01–4.89 3.61 (0.51)
Birth order: *n* (%)			
First-born	26 (72)	13 (68)	13 (77)
Maternal Age			
Range *M* (*SD*)	26–41 32.69 (4.05)	26–40 32.74 (3.57)	26–41 32.65 (4.64)
Maternal education			
Mean rank		15.42	21.94
Household income			
Mean rank		15.58	21.76

*M*: Mean, *SD:* Standard Deviation.

**Table 2:** Spearman correlations (ρ) of response time and vocabulary of 18-month-old children and mothers’ depression and anxiety scores.

Variable	Accuracy	Latency	Mean Depression	Mean Anxiety
OZI	0.594 ***	−0.609 **	−0.455 *	−0.351
Accuracy	1/1	−0.491 *	−0.538 **	−0.434 *
Latency		1/1	0.334	0.318
Depression			1/1	0.758 **

Note: Depression = Center for Epidemiologic Studies Depression Scale—Revised (CESD-R) scores; Anxiety = State-Trait Anxiety Inventory (STAI) scores; OZI = Australian-English Communicative Development Inventory; * *p* < 0.05; ** *p* < 0.01; *** *p* < 0.001.

**Table 3:** Hierarchical linear regression models with proportion of looking time to target post-naming (Model 1) and latency of gaze switch to target (Model 2) as dependent variables.

Model 1: Proportion of Looking Time to Target								
Removed covariate		*R^2^* = 0.389, *F*(2, 28) = 8.930, *p* = 0.001						
		*β*		*SE*		*T*		*P*
Vocabulary size		0.327				2.035		0.052
**Entered predictor variables**								
Mean postnatal depression		−0.677		0.006		−3.180		0.004 ***
Mean postnatal anxiety		0.076		0.004		0.358		0.723
**Model 2: Latency of Gaze Switch to Target**								
**Removed covariate**	***R^2^* = 0.286, *F*(2, 18) = 3.609, *p* = 0.048**							
	***β***		***SE***		*T*		***P***	
Vocabulary size	−0.383		0.328		−1.798		0.090	
**Entered predictor variables**								
Mean postnatal depression	0.399		5.584		1.429		0.170	
Mean postnatal anxiety	0.173		4.234		0.618		0.544	

Note. *β* = standardized regression co-efficient; SE = standard error; T = test statistic; P = probability; Depression = CESD-R scores; Anxiety = STAI scores; *** *p* < 0.001

## Supplementary Materials

The following are available online at https://www.mdpi.com/2076-3425/10/12/977/s1, Table S1: Maternal depression and anxiety scores in the control and risk groups; Table S2: Acoustic analysis of auditory stimuli.
