# Supplementary material for: Maternal Depression Affects Infants’ Lexical Processing Abilities in the Second Year of Life
Source: Brain Sci. 2020 Dec 12;10(12):977. doi: 10.3390/brainsci10120977 (PMC7763905; doi:10.3390/brainsci10120977)
Supplement: Supplementary file 1 [file brainsci-10-00977-s001.pdf]

Supplementary Files

Table S1. Maternal depression and anxiety scores in the control and risk groups.

Descriptive statistics		Group				
		**Control**	**Risk**	***t***	***df***	***p***
6-month assessment		*n* = 18	*n* = 15			
Depression	Range	0 - 11	0 - 28			
	Mean (SD)	4.67(3.48)	8.87 (7.59)	-2.103	31	.044
	%	0	20			
Anxiety	Range	21 - 39	22 - 47			
	Mean (SD)	29.61 (6.02)	31.80 (8.091)	-.890	?	.380
	%	0	15			
9-month assessment		*n* = 21	*n* = 19			
Depression	Range	0 - 13	0 - 21			
	Mean (SD)	4.71 (4.27)	9.00 (7.71)	-2.144	27.46	.041
	%	0	32			
Anxiety	Range	20 - 46	21 - 55			
	Mean (SD)	29.62 (6.76)	35.63 (9.06)	-2.393	38	.022
	%	5	32			
12-month assessment		*n* = 24	*n* = 19			
Depression	Range	0 - 13	0 - 27			
	Mean (SD)	4.54 (4.02)	10.26 (8.02)	-2.840	25.10	.009
	%	0	26			
Anxiety	Range	21 - 37	20 - 51			
	Mean (SD)	27.88 (4.76)	36.00 (9.27)	-3.474	25.42	.002
	%	0	37			
18-month assessment		*n* = 23	*n* = 20			
Depression	Range	0 - 20	0 - 26			
	Mean (SD)	5.09 (4.60)	9.20 (8.46)	-4.113	28.41	.062
	%	4	20			
Anxiety	Range	20 - 42	20 - 62			
	Mean (SD)	28.39 (6.65)	34.58 (11.48)	-2.111	29.54	.043
	%	4	25			
Mean postnatal score		*n* = 25	*n* = 21			
Depression	Range	0 - 13	0 - 22.5			
	Mean (SD)	4.63 (3.61)	9.22 (6.97)	-2.685	26.81	.012
Anxiety	Range	20.75 - 39.67	21 - 45			
	Mean (SD)	28.32 (4.91)	34.58 (7.56)	-3.221	30.85	.003

Note. Depression = CESD-R scores; Anxiety = STAI scores; % = the percentage of mothers with scores at or above the clinical threshold. The mean depression and anxiety scores differ from the cut off scores described in the group allocation criteria as they reflect the average score obtained across the four assessment time points.

Table S2. Acoustic analysis of auditory stimuli.

Audio stimuli	F0 Mean	F0 Minimum	F0 Maximum	Target word Duration (Ss)
Look at the ball	336.20	168.79	480.09	0.55
Look at the book	315.29	175.04	523.88	0.47
Look at the car	351.04	163.61	489.15	0.58
Look at the cup	332.74	175.71	522.07	0.46
Look at the hat	330.45	194.33	502.10	0.56
Look at the shoe	312.72	147.73	492.46	0.62
Where is the ball	304.13	79.32	447.90	0.61
Where is the book	315.40	205.26	515.13	0.44
Where is the car	287.85	148.85	423.22	0.65
Where is the cup	355.72	214.39	524.28	0.40
Where is the hat	362.54	216.69	511.25	0.60
Where is the shoe	322.11	163.12	439.58	0.62
Filler-Look	312.03	184.15	526.11	NA
Filler-Wow	257.28	173.89	355.55	NA

Note. Ss = Seconds.
